# Supplementary material for: Overexpression of Ssd1 and calorie restriction extend yeast replicative lifespan by preventing deleterious age-dependent iron uptake
Source: bioRxiv. 2025 Sep 3:2025.09.02.673772. Preprint. [Version 1] doi: 10.1101/2025.09.02.673772 (PMC12424809; doi:10.1101/2025.09.02.673772)
Supplement: Supplementary file 1 [file NIHPP2025.09.02.673772v1-supplement-1.pdf]

bioRxiv preprint doi: https://doi.org/10.1101/2025.09.02.673772; this version posted September 3, 2025. The copyright holder for this preprint (which was not certified by peer review) is the author/funder, who has granted bioRxiv a license to display the preprint in perpetuity. It is made available under aCC-BY 4.0 International license.

Supplemental Movie 1. Bright field (**A.**) and corresponding fluorescence microscopy (**B.**) of Ssd1-GFP for an SSD1-GFP cell during the RLS. Bright field (**C.**) and corresponding fluorescence microscopy (**D.**) of Ssd1-GFP for a P_{GPD1}-SSD1-GFP cell during the RLS. Numbers indicate the number of cell divisions into the lifespan.

Supplemental Movie 2. Fluorescence microscopy of Ssd1-GFP condensates undergoing fusion in an aging P_{GPD1}-SSD1-GFP cell.

Supplemental Table 1: Yeast strains used in this study

IGY138	MATα *his3Δ leu2Δ ura3Δ ssd1*::NatMX
IGY081	MATα *his3Δ leu2Δ ura3Δ SSD1*-GFP (KanMX6)
IGY086	MATα *his3Δ leu2Δ ura3Δ* P_{GPD1}-*SSD1*-GFP (KanMX6)
IGY536	MATα *his3Δ leu2Δ ura3Δ SSD1*-mCherry (*HIS3*)
IGY540	MATα *his3Δ leu2Δ ura3Δ* P_{GPD1}-*SSD1*-mCherry (*HIS3*)
IGY106	MATα *his3Δ leu2Δ ura3Δ SSD1*-GFP (KanMX6) *PAB1*-mCherry (*HIS3*)
IGY115	MATα *his3Δ leu2Δ ura3Δ* P_{GPD}-1*SSD1*-GFP (KanMX6) *PAB1*-mCherry (*HIS3*)
IGY107	MATα *his3Δ leu2Δ ura3Δ SSD1*-GFP (KanMX6) *EDC3*-mCherry (*HIS3*)
IGY110	MATα *his3Δ leu2Δ ura3Δ* P_{GPD1}-*SSD1*-GFP (KanMX6) *EDC3*-mCherry (*HIS3*)
IGY117	MATα *his3Δ leu2Δ ura3Δ SSD1*-GFP (KanMX6) *HSP104*-mCherry (*HIS3*)
IGY111	MATα *his3Δ leu2Δ ura3Δ* P_{GPD1}-*SSD1*-GFP (KanMX6) *HSP104*-mCherry (*HIS3*)
IGY544	MATα *his3Δ leu2Δ ura3Δ SSD1*-mCherry (HIS3) URA3-P_{GPD1}-GFP_*AFT1*
IGY545	MATα *his3Δ leu2Δ ura3Δ* P_{GPD1}-*SSD1*-GFP-mCherry (*HIS3*) *URA3*-P_{GPD1}-GFP_*AFT1*
IGY521	MATα *his3Δ leu2Δ ura3Δ SSD1*-GFP (KanMX6) *ARN1*-mCherry (*HIS3*)
IGY522	MATα *his3Δ leu2Δ ura3Δ* P_{GPD1}-*SSD1*-GFP (KanMX6) *ARN1*-mCherry (*HIS3*)
IGY529	MATα *his3Δ leu2Δ ura3Δ SSD1*-GFP (KanMX6) *FIT2*-mCherry (*HIS3*)
IGY530	MATα *his3Δ leu2Δ ura3Δ* P_{GPD1}-*SSD1*-GFP (KanMX6) *FIT2*-mCherry (*HIS3*)
IGY516	MATα *his3Δ leu2Δ ura3Δ SSD1*-GFP (KanMX6) *aft1*::*HIS3*

bioRxiv preprint doi: https://doi.org/10.1101/2025.09.02.673772; this version posted September 3, 2025. The copyright holder for this preprint (which was not certified by peer review) is the author/funder, who has granted bioRxiv a license to display the preprint in perpetuity. It is made available under aCC-BY 4.0 International license.

IGY524	MATα *his3Δhis3Δ leu2Δ ura3Δ* P_{GPD1}-*SSD1*-GFP (KanMX6) *aft1*::*HIS3*
